# Anterior shift of the ventral dura mater: A novel concept of the posterior surgery for ossification of the posterior longitudinal ligament in thoracic spine

**DOI:** 10.3389/fsurg.2023.1120069

**Published:** 2023-04-11

**Authors:** Kohei Takahashi, Ko Hashimoto, Takahiro Onoki, Haruo Kanno, Hiroshi Ozawa, Toshimi Aizawa

**Affiliations:** ^1^ Department of Orthopaedic Surgery, School of Medicine, Tohoku University, Sendai, Japan; ^2^ Department of Orthopaedic Surgery, School of Medicine, Tohoku Medical and Pharmaceutical University, Sendai, Japan

**Keywords:** ossification of the posterior longitudinal ligament, thoracic spine, dura mater, anterior decompression, myelopathy

## Abstract

**Background:**

Thoracic myelopathy caused by ossification of the posterior longitudinal ligament (OPLL) remains one of the most difficult disorders to treat. The Ohtsuka procedure, extirpation, or anterior floating of the OPLL through a posterior approach, has shown great surgical outcomes after several modifications. However, these procedures are technically demanding and pose a significant risk of neurological deterioration. We have developed a novel modified Ohtsuka procedure in which removal or minimization of the OPLL mass is unnecessary; instead, the ventral dura mater is shifted anteriorly with the posterior part of the vertebral bodies and targeted OPLL.

**Surgical Procedure:**

First, pedicle screws were inserted at more than three spinal levels above and below the spinal level where pediculectomies were performed. After laminectomies and total pediculectomies, partial osteotomy of the posterior vertebra adjacent to the targeted OPLL was performed by using a curved air drill. Then, the PLL is completely resected at the cranial and caudal sites of the OPLL using special rongeurs or a threadwire saw with a diameter of 0.36 mm. The nerve roots were not resected during surgery.

**Methods:**

Eighteen patients (follow-up ≥1 year) treated with our modified Ohtsuka procedure were assessed clinically, including the Japanese Orthopaedic Association (JOA) score for thoracic myelopathy and radiographically.

**Results:**

The average follow-up period was 3.2 years (range, 1.3–6.1 years). The preoperative JOA score was 2.7 ± 1.7, which improved to 8.2 ± 1.8 at 1 year postoperatively; therefore, the recovery rate was 65.8 ± 19.8%. The CT scan at 1 year after surgery revealed the anterior shift of the OPLL averaged 3.1 ± 1.7 mm and the ossification-kyphosis angle of the anterior decompression site decreased at an average of 7.2 ± 6.8 degrees. Three patients demonstrated temporary neurological deterioration, all of whom completely recovered within 4 weeks postoperatively.

**Discussion:**

The concept of our modified Ohtsuka procedure is 1) not OPLL extirpation or minimization but only the creation of space between the OPLL and spinal cord by an anterior shift of the ventral dura mater, which is achieved by complete resection of the PLL at the cranial and caudal sites of the OPLL; and 2) no nerve roots are sacrificed to prevent ischemic spinal cord injury. This procedure is not technically demanding and safe and provides secure decompression for thoracic OPLL. The anterior shift of the OPLL was smaller than expected, but it resulted in a relatively good surgical outcome with a recovery rate ≥65%.

**Conclusion:**

Our modified Ohtsuka procedure is quite secure and is not technically demanding, with a recovery rate of 65.8%.

## Introduction

In our previous epidemiological study in the Japanese population, 21% of thoracic myelopathy is caused by ossification of the posterior longitudinal ligament (OPLL) with or without ossification of the ligamentum flavum (1), which remains one of the most difficult-to-treat disorders for spinal surgeons. The difficulty of surgery depends on two reasons: anatomical features and perioperative complications. Anatomically, the spinal cord has a particularly vulnerable region, usually between T4 and T9, called the “watershed zone” with poor blood supply (2, 3). In addition, the thoracic spine is naturally kyphotic, and decompressive laminectomy is less effective because of the restricted postoperative backward shift of the spinal cord (4). From the viewpoint of perioperative complications, the multi-center studies from the Japanese Society for Spine Surgery and Related Research reported complication rates were 40.8‒51.3%, including 11.7‒26.3% of postoperative neurological deterioration (5–8).

Many surgical procedures have been developed for patients with thoracic OPLL to ensure more stable and effective outcomes. Since the report by Yamazaki et al. in (4), posterior decompression with instrumented spinal fusion (PDF) has been the gold standard, and approximately 75% of patients in Japan are now treated using it because of its safety and effectiveness (7). PDF has shown relatively good surgical outcomes (4–7). Meanwhile, theoretically, extirpation or anterior floating of the OPLL should allow for a more effective decompression of the spinal cord than PDF. However, those anterior decompression have a certain risk of postoperative paralysis despite their approaches, through a thoracotomy or a posterior approach (5, 9). We have produced a modified procedure of anterior decompression through a posterior approach based on a novel concept, in which extirpation or minimization of the OPLL mass is not necessary; instead, an anterior shift of the ventral dura mater (not anterior shift of the spinal cord) is achieved by complete resection of the PLL at the cranial and caudal sites of the targeted OPLL (10–12). In the present study, we introduced our surgical technique and demonstrated the surgical outcomes in 18 patients treated with this procedure.

## Patients and methods

### Our surgical procedure

**Figure 1:**
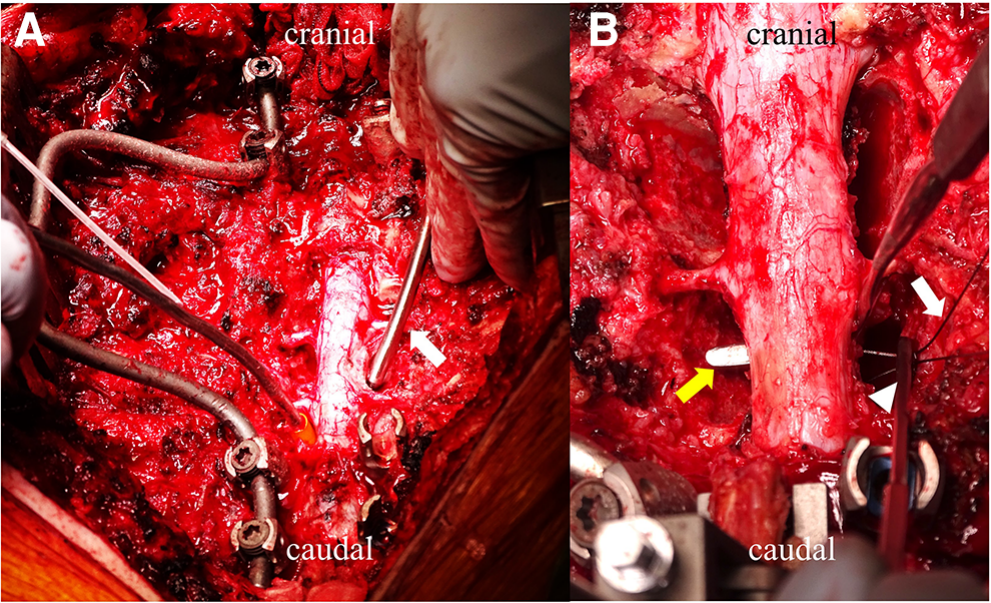
Intraoperative photographs of our modified ohtsuka procedure. (**A**) Using a curved air drill (white arrow), partial osteotomy of the vertebral bodies adjacent to the targeted ossification of the posterior longitudinal ligament (OPLL) is performed to make a “bone tunnel” at the posterior part of the vertebral bodies. (**B**) The posterior longitudinal ligament is cut at the cranial side of the OPLL by a threadedwire saw (white arrow) with a knot pusher (arrowhead) used under an angled spatula (yellow arrow).

**Figure 2:**
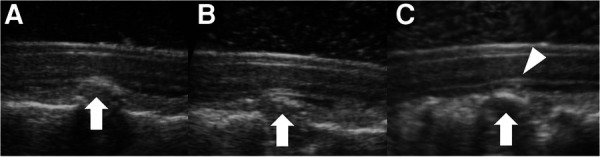
Intraoperative ultrasonography. After laminectomy. (**A**) The spinal cord is compressed anteriorly by ossification of the posterior longitudinal ligament (OPLL) (arrow). (**B**) Before resection of the PLL. After partial osteotomy of the posterior vertebra adjacent to the targeted OPLL and before PLL resection, the OPLL decreased (arrow). (**C**) After PLL resection. The OPLL further moves anteriorly (**arrow**), and a space between the spinal cord and dura mater is clearly detected (**arrowhead**).

**Figure 3:**
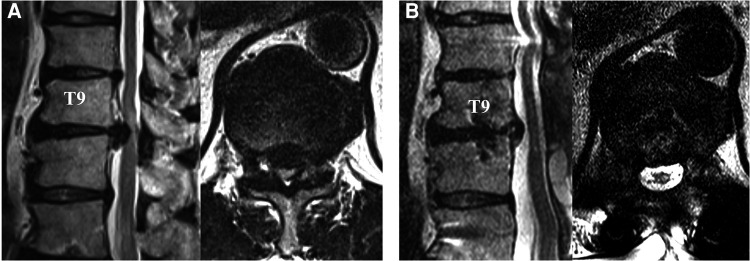
Pre- (**A**) and postoperative MRI (**B**) of the patient, a 45-year-old male, with ossification of the posterior longitudinal ligament (OPLL) at T9–10. (**A**) The spinal cord is severely compressed from anteriorly by OPLL at T9–10 with significant T2 high signal intensity. (**B**) After our modified Ohtsuka procedure, the spinal cord was completely decompressed, and cerebrospinal fluid was confirmed at the ventral side of the spinal cord.

The detailed surgical procedure can be found in our previous article, with a video presentation (12). A summary of the surgical procedure is presented here. This procedure was performed using spinal cord monitoring. Under general anesthesia, the patient was placed in the prone position. First, pedicle screws were inserted at more than three levels above and three below the spinal levels where pediculectomies were performed. After laminectomies and total pediculectomies, partial osteotomy of the posterior vertebra adjacent to the targeted OPLL was performed by using a curved air drill. Then, the PLL was completely resected at the cranial and caudal sites of the OPLL using special rongeurs or a threadwire saw with a diameter of 0.36 mm (13). In order to resect the PLL safely and surely, we determined the extension of pediculectomies so that no OPLL was confirmed on preoperative computed tomography (CT) at the site of PLL resection. This manipulation shifted the ventral dura mater anteriorly, together with the OPLL and the posterior part of the vertebral bodies. Finally, the final rods were connected to the pedicle screws. The intraoperative photographs, ultrasonography, and pre- and postoperative imaging findings are shown in Figures 1–3.

### Patients

From 2015 to 2021, we performed our modified Ohtsuka procedure in 21 patients with thoracic myelopathy caused by OPLL. Among them, two patients with a history of previous spinal surgery, one patient with intellectual disability were excluded from the analysis. Subsequently, 18 patients who were followed up for ≥1 year were included in this study.

### Clinical evaluations

From the operative records, the total number of vertebrae where pediculectomies were performed was counted (e.g., when pediculectomies were performed in T7, T8 and T9, “the total number of vertebrae where pediculectomies were performed” was recorded as 3). In addition, the operative duration, intraoperative blood loss, and changes in intraoperative spinal cord monitoring results were confirmed. Each patient's neurological condition was evaluated using the modified Japanese Orthopaedic Association (JOA) score, an 11-point scale measuring lower motor function, sensory function in the lower extremities and trunk, and bladder function (14). The modified Frankel scale was used to assess walking ability (15, 16). On this scale, Grade A is defined as “complete motor and sensory loss,” Grade B is defined as “preserved sensory only,” Grade C is defined as “preserved motor loss less than fair grade,” and Grade E is defined as “normal.” Grade D was classified into three subgrades: D1, D2, and D3. D1 is defined as “preserved motor ability at the lowest functional grade and ability to walk 10 to 100 m.” Patients usually use a wheelchair. D2 is defined as “preserved motor ability at midfunctional grade and ability to walk stably with a cane, handrail, and/or lower leg brace.” D3 is defined as “preserved motor ability at high functional grade and ability to walk without any support.” The neurological condition and walking abilities were evaluated preoperatively and one year after surgery because at one year postoperatively, the condition of patients became stable. Perioperative complications were also noted. Neurological data and complications were collected from the medical records.

### Radiographical evaluations

**Figure 4:**
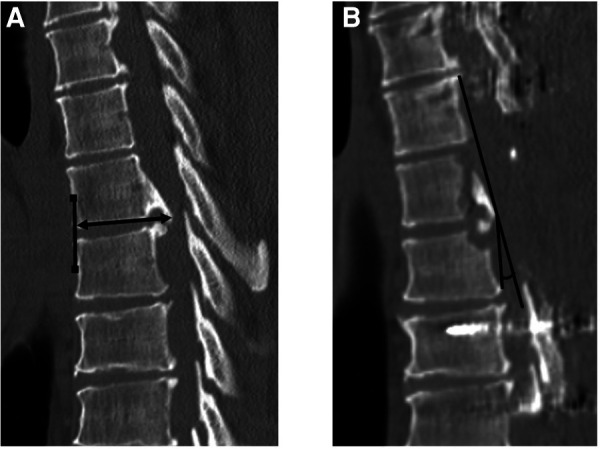
Definition of measurements of ossification of the posterior longitudinal ligament (OPLL) distance (**A**) and ossification-kyphosis angle (**B**). (**A**) The OPLL distance was defined as the distance between the prominence of the OPLL and the anterior margin of the vertebral bodies. The anterior margin was defined as the line connecting the midpoints of the upper and lower vertebral bodies of the corresponding OPLL. (**B**) The ossification-kyphosis angle was defined according to Tokuhashi et al. (18). This is the angle from the superior margin at the cranial vertebral body of the decompression site and from the lower posterior margin at the caudal vertebral body of the decompression site to the prominence of the maximum OPLL.

Spinal levels and length of the OPLL were confirmed. The type of OPLL was classified as linear, beaked, continuous waveform, continuous cylindrical, or mixed (composed of at least two of these types) according to the annual report of the taskforce of research for ossification of spinal ligaments sponsored by the Japanese Ministry of Health and Welfare in 1994 (17). Using sagittal reconstructed CT before and one year after surgery, the distance between the prominence of the OPLL and the anterior margin of the vertebral body (OPLL distance) was measured. In addition, the anterior shift of the prominence of the OPLL was defined as the difference between the pre- and postoperative OPLL distances (Figure 4A). The ossification-kyphosis angle at the anterior decompression site was gauged. This angle was based on Tokuhashi's definition: the angle from the superior margin at the cranial vertebral body of the decompression site and from the lower posterior margin at the caudal vertebral body of the decompression site to the prominence of the maximum OPLL (Figure 4B) (18). The difference between the pre- and postoperative ossification-kyphosis angles was also calculated.

### Statistical analysis

Statistical analysis was performed using the unpaired t-test for the JOA score, OPLL distance, and ossification-kyphosis angle compared to preoperative data with those one year after surgery. All collected data were analyzed using commercially available software (JMP Pro®. (SAS Institute Inc., Cary, NC, USA). Statistical significance was set at *P* < 0.05.

## Results

Eight males and ten females were included in the study, and the average age at surgery was 50 years (range, 36–74 years). The average follow-up period was 3.2 years (range, 1.3‒6.1 years). The demographic data of each patient are presented in Table 1. All the patients had thoracic myelopathy. The average total number of vertebrae where pediculectomies were performed was 2.9 (range, 2‒5). Fifteen patients underwent fewer than three vertebral pediculectomies, while three patients underwent more than four levels of pediculectomy. Operative duration and intraoperative blood loss are presented in Table 2.

**Table 1:** Demographic data of the patients.

Age at surgery (years)	50 ± 11
Sex	Male : female = 8 : 10
Body Mass Index (kg/m^2^)	30.6 ± 6.3
Diabetes mellites	4/18
Follow-up period (years)	3.2 ± 1.5

The numeric values are shown as mean ± standard deviation.

**Table 2:** Intraoperative data of our modified ohtsuka procedure.

Number of vertebrae with pediculectomies	2.9 ± 0.8
Operative duration (min)	577 ± 131
Intraoperative blood loss (ml)	1184 ± 707
**Motor evoked potentials (MEPs)**	**17/18**
Detected preoperatively	9/17
Disappeared intraoperatively	1/9
≥70% decrease intraoperatively	4/9

The numeric values are shown as mean ± standard deviation.

The changes in the intraoperative spinal cord monitoring results are shown in Table 2. Intraoperative neurophysiological monitoring was performed on 17 patients. Motor evoked potentials (MEPs) were detected in 9 patients before surgery. During surgery, MEPs of one patient disappeared, and those of 4 patients showed a ≥70% decrease.

Neurological evaluation results are shown in Table 3. The preoperative JOA score was 2.7 ± 1.7 (mean ± standard deviation), which significantly improved to 8.2 ± 1.8 one year after surgery. The recovery rate was 65.8 ± 19.8% (14). Using the modified Frankel scale before surgery, non-ambulatory patients accounted for 55.6% before surgery. One year postoperatively, one patient showed four grade-ups, five patients showed three grade-ups, four patients showed two grade-ups, and eight patients showed one grade-up in walking ability. All preoperative non-ambulatory patients could at least walk using support such as crutches and a cane one year after surgery. Three patients demonstrated temporary neurological deterioration, all of whom completely recovered within 4 weeks postoperatively.

**Table 3:** Pre- and postoperative neurological evaluation of the patients.

Preop. JOA score	2.7 ± 1.7
Postop. JOA score	8.2 ± 1.8
Recovery rate (%)	65.8 ± 19.8
**Change of modified Frankel scale**	
B to D2	1
C to D1	1
C to D2	4
C to D3	3
C to E	1
D1 to D2	2
D1 to E	1
D2 to D3	4
D3 to E	1

The numeric values are shown as mean ± standard deviation.

JOA score: Japanese Orthopaedic Association score for thoracic myelopathy (full mark: 11) [16].

Recovery rate (%) = (Postop. JOA score-Preop. JOA score)/11-Preop. JOA score × 100.

Modified Frankel scale.

B: preserved sensory only.

C: preserved motor loss less than fair grade.

D1: preserved motor ability at lowest functional grade. Usually uses a wheelchair.

D2: preserved motor ability at midfunctional grade. Usually uses a cane.

D3: Preserved motor ability at a high functional grade. No support was required for walking.

E: normal.

The radiographic evaluation results are shown in Table 4. The OPLL distance and ossification-kyphosis angle were significantly smaller postoperatively (*P* < 0.001). The anterior shift of the prominence of OPLL was 3.1 ± 1.7 mm, and the change of the ossification-kyphosis angle was 7.2 ± 6.8 degrees.

**Table 4:** Radiographic evaluation of the patients.

**Levels of OPLL**	
Upper	7
Middle	9
Lower	2
Length of OPLL (number of vertebrae)	2.9 ± 1.0
**Type of OPLL** (17)	
Linear	0
Beaked	4
Continuous waveform	1
Continuous cylindrical	2
Mixed	11
Including beaked type	15
OLF at the narrowest segment	13
Preoperative OPLL distance	31.7 ± 4.7
Postoperative OPLL distance	28.5 ± 5.5
Preoperative ossification-kyphosis angle	24.2 ± 6.4
Postoperative ossification-kyphosis angle	17.0 ± 5.3

The length of the OPLL, OPLL distance, and ossification-kyphosis angle are shown as the mean ± standard deviation.

Upper: T1–5, middle: T5–8, lower: T8–12.

Mixed: composed of at least two of either linear, beaked, continuous waveforms, or continuous cylindrical type.

OLF: ossification of the ligamentum flavum.

OPLL distance: the distance between the prominence of the OPLL and the anterior margin of the vertebral body.

Ossification-kyphosis angle: The angle between the line joining the superior margin of the cranial end of the laminectomized vertebral body and the maximal prominence of the OPLL and the line joining the inferior margin of the caudal end of the laminectomized vertebral body and the maximal prominence of the OPLL.

## Discussions

Thoracic OPLL was first reported by Forcier et al. in (19). A 68-year-old male underwent laminectomy and achieved slight improvement. Thereafter, many spine surgeons have challenged this intractable disease with many kinds of procedures, including laminectomy with or without resection of the dentate ligaments (1, 20), anterior decompression and fusion through a thoracotomy (21, 22), posterior decompression with instrumented spinal fusion (4, 23), and anterior decompression through a posterior approach, the so-called Ohtsuka procedure (24). More specific procedures include circumspinal decompression through a posterior approach with thoracotomy (25), through a single posterior approach (9) and a posterolateral approach (26), staged spinal decompression through a posterior approach (27), and single-stage mini-thoracotomy without spinal fusion (3, 28).

Since the thoracic spine is usually kyphotic and OPLL compresses the spinal cord anteriorly; theoretically, extirpation or anterior floating of the OPLL should allow for more effective decompression of the spinal cord (4, 10, 11). Two surgical approaches can be used for this purpose: thoracotomy and a single posterior or posterolateral approach. These procedures have reported good surgical outcomes but might be technically demanding (5–7, 9); Kato et al. (24). The rate of postoperative neurological deterioration is higher (4–7, 9). In addition, anterior decompression through thoracotomy has a significant risk of pulmonary complications, including cerebrospinal fluid leakage into the thoracic cavity (3, 5).

The concept of our modified Ohtsuka procedure is completely different from that of other procedures. The goal is not OPLL extirpation but only the creation of space between the OPLL and spinal cord by an anterior shift of the ventral dura mater. Minimization of the OPLL mass was not necessary. Instead, complete resection of the PLL at the cranial and caudal sites of the OPLL, where no OPLL is confirmed on preoperative CT, is required. Since the ventral dura mater severely adheres to the OPLL, occasionally, ossified dura mater is detected. While the spinal cord does not cling to the dura mater, the ventral dura should shift anteriorly, pulled by the ossified mass, after anterior decompression of the OPLL with cranial and caudal PLL resection. Therefore, the procedure is not technically demanding. Invasion to the spinal cord should be less than OPLL extirpation or minimization. Recently, a similar concept of indirect decompression surgery was reported by Ma et al. (29) as thoracic column antedisplacement and fusion, with a mean recovery rate of 42.3%.

In the present study, the anterior shift of the prominence of OPLL was small, only 3.1 mm, and the change in the ossification-kyphosis angle was less than 10 °. These values may be smaller than expected; however, the postoperative outcomes were excellent: the recovery rate was more than 65%, which was better than that of the PDF and compared favorably with the other modified Ohtsuka procedures (4, 7, 9, 30, 31). It should be led by a secure anterior shift of the targeted OPLL achieved by resecting all four sides of the OPLL from the surrounding tissues, creating a significant space between the ventral dura mater and the spinal cord.

There are two advantages of our modified Ohtsuka procedure. First, because a curved air drill was used, no nerve roots were sacrificed. Therefore, ischemic spinal cord injury should be prevented as blood supply to the spinal cord is preserved. Second, extirpation or anterior floating of the OPLL carries a potential risk of spinal cord damage because the targeted OPLL usually severely compresses the spinal cord. These manipulations may further damage the weakened spinal cord even if a surgical microscope is used. In our procedure, extirpation or minimization of the OPLL mass is not performed, and the PLL is resected where no OPLL can be detected, not at the narrowest spinal level, usually done by a very fine T-saw with a diameter of 0.36 mm (13). Invasion of the spinal cord should be reduced. Although several pathologies may be out of indication, such as continuous OPLL from the cervical to the thoracic spine and a very long OPLL from T1 to T11, this procedure is less technically demanding, safer, and offers secure decompression.

The present study confirmed the significantly good surgical outcomes of our modified Ohtsuka procedure in patients with thoracic OPLL; however, several limitations should be noted. The study population was limited because thoracic OPLL is a rare condition. The outcome could be compared with that of previous reports. A randomized prospective comparison between this procedure and other surgical methods with a sufficient number of patients is ideal, but it should be difficult because of the small number of patients, in addition to ethical reasons. We should increase the number of patients and verify the safety and effectiveness of this procedure in a larger population.

## Data Availability

The raw data supporting the conclusions of this article will be made available by the authors, without undue reservation.
